# Maternal bisphenols exposure and thyroid function in children: a systematic review and meta-analysis

**DOI:** 10.3389/fendo.2024.1420540

**Published:** 2024-07-01

**Authors:** Jiani Liu, Min Tian, Haiyue Qin, Danrong Chen, Sabitina Mrisho Mzava, Xu Wang, Francis Manyori Bigambo

**Affiliations:** ^1^ JC School of Public Health and Primary Care, Faculty of Medicine, The Chinese University of Hong Kong, Hong Kong, Hong Kong SAR, China; ^2^ Department of Gastroenterology, Children’s Hospital of Nanjing Medical University, Nanjing, China; ^3^ Nanjing Foreign Language School, Nanjing, China; ^4^ School of Public Health, Nanjing Medical University, Nanjing, China; ^5^ Department of Nephrology, Muhimbili National Hospital, Dar es Salaam, Tanzania; ^6^ Clinical Medical Research Center, Children’s Hospital of Nanjing Medical University, Nanjing, China

**Keywords:** bisphenols, prenatal exposure, children, thyroid function, meta-analysis

## Abstract

**Background:**

Evidence from animal experiments and epidemiological studies has reported controversial results about the effects of prenatal bisphenols (BPs) exposure on childhood thyroid function. This study aims to explore the associations of prenatal exposure to BPs with thyroid-related hormones (THs) in newborns and early childhood, with a particular focus on the sex-dependent and exposure level effects.

**Methods:**

Correlated studies were systematically searched from PubMed, Web of Science, Medline, Cochrane, and Embase until February 21, 2024. The exposures assessed include bisphenol A (BPA), bisphenol F (BPF), bisphenol S (BPS), bisphenol AF (BPAF), and tetrachlorobisphenol A (TCBPA). THs measured were thyroid stimulating hormone (TSH), total tri-iodothyronine (TT3), total thyroxine (TT4), free tri-iothyronine (FT3), and free thyroxine (FT4). Effect estimates were quantified using coefficients from multivariable regression models. Statistical analyses were completed using Stata 16.0. The methodological quality of the included studies was evaluated using the Newcastle-Ottawa Scale (NOS).

**Results:**

Eleven cohort studies comprising 5,363 children were included in our meta-analysis. Prenatal bisphenol concentrations were statistically significant related to alterations in thyroid hormones in children, exclusively in female offspring, including reduced TSH (β = -0.020, 95% CI: -0.036, -0.005) and increased TT3 levels (β = 0.011, 95% CI: 0.001, 0.021), and exposure to high concentration of bisphenols (>1.5 ug/g creatinine) significantly reduced FT3 levels in children (β = -0.011, 95% CI: -0.020, -0.003).

**Conclusion:**

Prenatal bisphenol exposure is linked to alterations in thyroid hormone levels in girls, necessitating enhanced measures to control bisphenol exposure levels during pregnancy for child health protection.

**Systematic Review Registration:**

https://inplasy.com, identifier INPLASY202450129.

## 1 Introduction

Bisphenols (BPs) are integral to plasticizers in the synthesis of polycarbonate plastics and epoxy resins (1–5). They are pervasive in the manufacturing of industrial, agricultural, and personal care products (6), such as toys, beverage containers, the interior coating of food cans, medical tubes, dental sealants, and water supply pipes (1, 3, 7–9). Bisphenols are found in urine samples from nearly all individuals undergoing medical examinations (10–12), as well as in samples of placenta, amniotic fluid, breast milk, and umbilical cords from pregnant women (2, 13–20). This suggests that BP exposure begins prenatally as it crosses the placental barrier, and continues across postnatal life (21). BPA is the most frequently utilized and extensively-studied contaminant. Low doses of BPA estrogenic activity and a high affinity for uterine tissue (22), and exposure of the fetus to BPA *in utero* might have more pronounced adverse effects, including poor sperm quality, abnormal menstrual cycle, adiposis, obvious neurobehavioral issues in children, and fluctuations in thyroid hormone levels and blood pressure. Considering the reported low metabolism rate of BPA in animal studies (23), the fetus might be particularly sensitive to BPA exposure (24), although this contaminant is present in low concentrations in the human body. Since the European Union, the United States, and China announced bans on the utilization of BPA in certain infant products in 2011, there has been a significant increase in the use of other bisphenol derivatives such as BPF, BPS, BPAF, and TCBPA as substitutes for BPA.

Thyroid hormones play complex roles in body growth and brain development both during prenatal and postnatal stages (1, 3). Thyroid hormones maintain homeostasis in our bodies, help synthesize proteins, maintain cardiovascular function, facilitate the growth and development of both the central nervous system and the skeletal system, as well as regulate hematopoiesis (5). Since BP is structurally similar to thyroid hormones (25), BPs may cause thyroid hormone disturbances (2, 26), which may affect thyroid-stimulating hormone (TSH), free and total triiodothyronine (FT3, TT3), or free and total thyroxine (FT4, TT4), by directly interacting with TH receptors acid. Previous researches have indicated that BPs interfere with the expression of genes related to thyroid function in both FRTL5 cells and zebrafish embryos by modulating the expression of thyroid transcription factors Foxe1, Nkx2-1, and Pax8 (27, 28). In addition, BPs also inhibit sodium/iodine transporters, thereby affecting thyroid hormone signaling and action (27). Multiple *in-vitro* studies and animal experiments suggested that interference with thyroid hormone action by BPs may adversely affect neurobehavioral outcomes and be related to the elevated potential for cognitive decline and children’s behavioral problems (29–32). BPA has been identified as capable of targeting the thyroid, leading to interference with its functions (33–35). Several *in vivo* and *in vitro* investigations, along with animal experiments, have delineated that specific concentrations of BPS and BPF can induce thyroid dysfunction by disrupting thyroid hormone synthesis (36) and perturbing endocrine equilibrium through modulation of gene transcription implicated in the hypothalamic-pituitary-thyroid (HPT) axis (28, 37). 7-day zebrafish embryo toxicity assays conducted by (38) have revealed a notable decrement in thyroid hormone receptor (TR) levels upon exposure to ≥12.5μg/L of BPAF, indicative of its potential as a TR antagonist (39). Furthermore, rodent studies (40, 41) and research involving amphibians (42) have postulated that TBBPA and TCBPA may serve as thyroid hormone disruptors. These findings underscore the plausible hazards posed by these compounds on thyroid function within organisms, warranting further investigation and vigilance within the scientific community.

In animal studies, elevated serum T4 levels were presented in female and male offspring after BPA exposure in pregnant mice (26, 43–45). In another study, FT4 levels increased only in male offspring (postnatal day 7) and then decreased after about two weeks (postnatal day 21) (44). There are also studies showing that prenatal exposure to BPA in pregnant rats did not affect offspring TSH and TT4 (1). In human studies, several prospective cohort studies have found that maternal urinary exposure to BPA is linked to lower TSH and TT4 among children, and a positive correlation with TT3 (1, 46). On the contrary, a cohort study conducted in China demonstrated a positive association between prenatal exposure to BPA or BPS with neonatal TSH levels, particularly pronounced in girls, while no significant correlation was detected between BPF exposure and thyroid hormone levels (47). A Netherlands cohort study showed that antenatal maternal high BPA exposure was only related to lower FT4 in six-year-old children, and also suggested that higher concentrations of BPF were correlated with higher levels of FT3. However, no significant effect of BPS was observed (48). Also, Fen Lin et al. confirmed that there is a negative association between BPA concentrations and FT3 in neonates (4), but American research indicated that there is no such relationship (2). Megan E. Romano et al. demonstrated that prenatal exposure to BPA did not affect any type of thyroid hormones (2). Studies conducted *in vivo* and *in vitro* have demonstrated that BPA halogenated derivatives exhibit similar or even greater endocrine toxicity than BPA, due to their structural similarity (49). Human exposure to environmental pollutants often involves simultaneous exposure to multiple substances rather than a single compound. In addition to investigating the relationship between individual bisphenol compound exposures and thyroid function, numerous studies have examined the collective impact of bisphenol mixtures on THs. A cohort study conducted in China unveiled a positive trend in the cumulative impact of six bisphenol compounds, including BPA, bisphenol B (BPB), bisphenol C (BPC), BPF, BPS, and BPAF, on FT4 and FT3 levels (50). Another study found significant correlations between maternal urinary bisphenol mixtures (BPA, BPF, BPS, BPAF, TCBPA) and TT3 concentration in newborn cord blood, with a slight but significant correlation with FT3 concentration (51). However, conflicting results also exist. Research by Arash Derakhshan et al. identified a link between prenatal bisphenols mixture exposure (BPA and BPS) and reduced FT4 levels in children, but found no correlation with TSH levels (48). The Bayesian kernel machine regression (BKMR) and restricted cubic spline (RCS) models revealed a non-linear correlation between bisphenol mixtures and FT3 (52).

Due to controversies in the findings of the previous studies, a meta-analysis was performed to investigate the effect of BPs as single-exposure and mixture exposure during pregnancy on the levels of TSH, TT3, TT4, FT3, and FT4 in children, particularly in the sex-specific manners.

## 2 Materials and methods

This systematic review and meta-analysis adhered strictly to the standard protocols outlined in the Cochrane Handbook and complied with the Preferred Reporting Items for Systematic Reviews and Meta-Analyses (PRISMA) guidelines (53). This meta-analysis was registered in INPLASY as INPLASY202450129, and no major amendments were made compared to the proposal.

### 2.1 Search strategy

Comprehensive literature searches were performed in PubMed, Web of Science, Medline, Cochrane, and Embase from the earliest available dates stated in each database until February 21, 2024. We employed a broad array of search terms to encompass various aspects of prenatal exposure and thyroid function, including “gestation”, “pregnancy”, “prenatal”, “antepartum”, “maternal”, “BPA”, “BPF”, “BPS” “bisphenol”, child” “kid”, “ped”, “neonatal”, “neonate”, “baby”, “newborn”, “infant”, “boy”, “girl”, “thyroid”, “TSH”, “TT3”, “TT4”, “FT3”, “FT4”, “total triiodothyronine”, “total thyroxine”, “free triiodothyronine”, “free thyroxine”, “thyroid-stimulating hormone”, “thyroid disease”, “hyperthyroidism”, “subclinical hyperthyroidism”, “hypothyroidism”, “subclinical hypothyroidism”. The detailed search terms are shown in **Supplementary Table 1**. Study search, data extraction, and quality assessment were conducted independently by two researchers, and discrepancies were resolved through group discussions until reach consensus.

### 2.2 Eligibility criteria

Studies were included if they (i) focused on prenatal bisphenols (BPs) exposure like (BPA, BPF, BPS, BPAF, and TCBPA and children’s thyroid function presented by thyroid hormone metric including TSH, TT3, TT4, FT3, and FT4); (ii) detected BPs from maternal blood, urine, amniotic fluid (AF), or umbilical cord blood samples; (iii) quantified measured the correlation between BP concentrations and TH levels in children. Studies were excluded if they were reviews, animal studies, conference abstracts, lectures, literature, or editorial materials, and if full texts were not available. After removing duplicates, the remaining literature was meticulously screened for relevance based on title, abstract, and full text, utilizing Endnote X20 for record management.

### 2.3 Data extraction

Two researchers extracted information from eligible papers using predefined templates independently. Data extracted include first author name, publication year, country/region, research design, number of children, bisphenol types measured, thyroid hormones measured, maternal age, reported effect estimates, the concentrations of bisphenols, and covariates adjusted in the analyses.

### 2.4 Quality assessment

The quality of each eligible study was assessed using the Newcastle Ottawa Scale (NOS). NOS consists of 3 categories (topic selection, comparability, and results) and 8 items. NOS scores range from 0 to 9 stars, with 4 stars allocated for selection, 2 stars for comparability, and 3 stars for results. A study is of high quality if the number of stars is greater than or equal to 6; medium quality if the number of stars is between 3 and 5; and low quality if the total number of stars is less than 3.

### 2.5 Statistical analysis

In our study, we investigated the effects of prenatal exposure to BPs on THs in total children and by sex. Subgroup analyses were performed based on types of thyroid hormones with all BPs included in the model. Furthermore, since BPA was predominantly reported among several eligible studies and highly controversial effects on THs existed, subgroup analyses of BPA and thyroid hormones were also performed. The effect sizes of eligible studies were synthesized using coefficients from multivariable regression models, alongside their 95% confidence intervals (CIs). In instances where studies reported percentage changes in thyroid hormone levels, conversions were performed to align with the common effect size metric (β represents the regression coefficient) as shown in the formula (2):


$$({\text{e}}^{\text{β}}-1)*100=\%\text{ change of THs}$$

Heterogeneity among studies was assessed using the I^2 statistic, with I^2^≥50% indicating considerable heterogeneity and necessitating a random-effects model. Conversely, a fixed-effects model was applied for I^2^<50%. Forest plots were employed to visually depict the results of the meta-analysis. Results were visually represented through forest plots, and subgroup analyses were conducted to explore the sex-specific and exposure level effects. There is no clear threshold for the concentration of prenatal bisphenols exposure that may cause thyroid dysfunction. We sorted all included studies by bisphenol concentrations and used the 50th percentile as the boundary to classify the included studies into low-exposure and high-exposure groups. To ascertain the stability of the results, sensitivity analyses were performed. Egger’s linear regression tests were used to check publication bias. The data acquisition and analysis processes were conducted utilizing Stata 16.0 software. Statistically significant was at p-value< 0.05.

## 3 Results

### 3.1 Literature search and study selection

**Figure 1:**
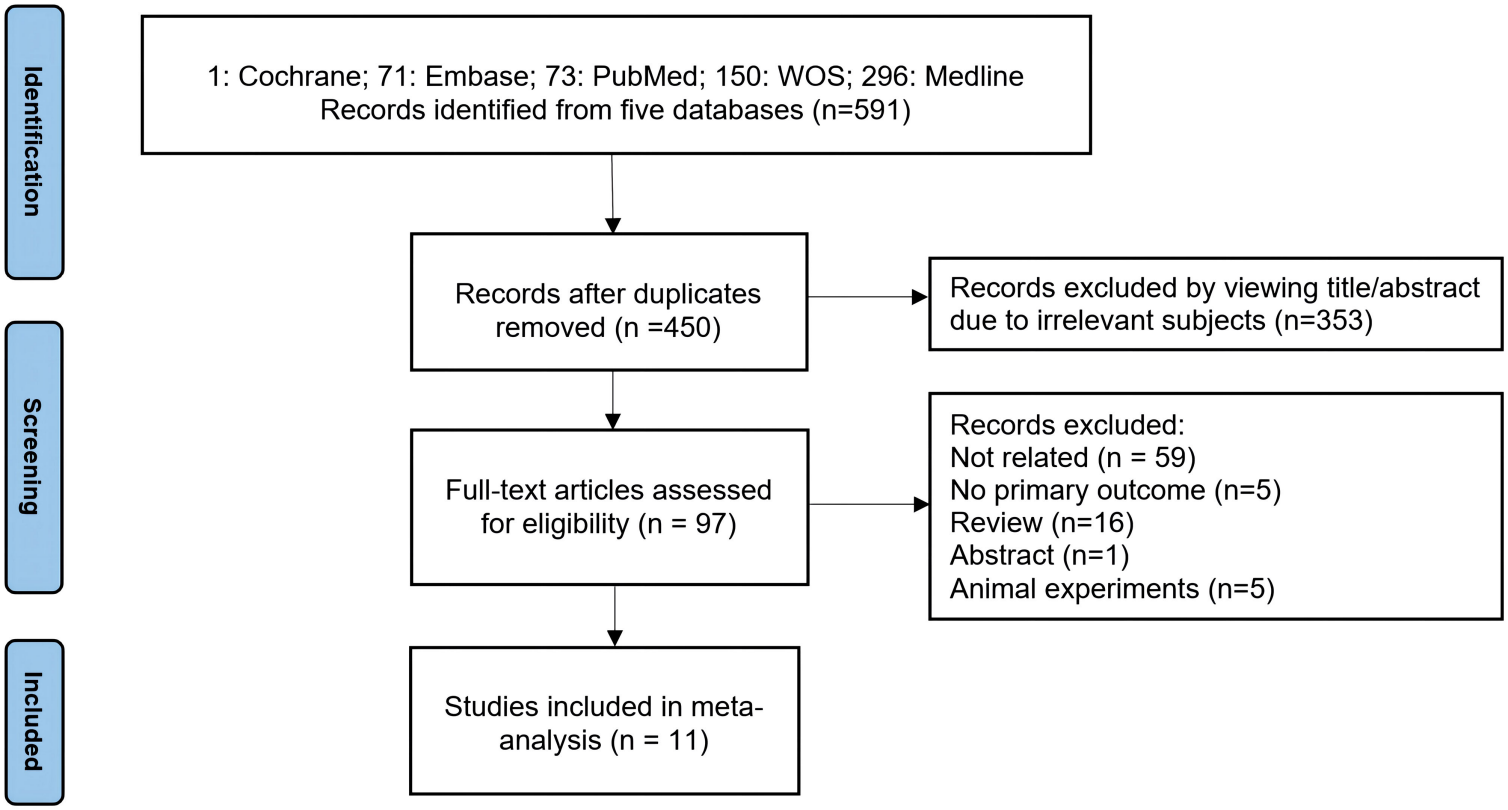
Flowchart of Literature Search.

An initial query across five major databases (PubMed, Web of Science, Medline, Cochrane, and Embase) retrieved 591 potential articles. After removing duplicates, the remaining 450 articles were screened by title and abstract resulting in 97 articles that were assessed in full text. This meticulous evaluation led to the exclusion of 59 articles due to irrelevance to the associations of prenatal BPs exposure with THs levels in children, 16 reviews, 5 animal studies, 1 abstract, and 5 articles lacking necessary data. As a result, 11 articles (1–5, 20, 48, 51, 54–56) were eligible for this meta-analysis (**Figure 1**).

### 3.2 Studies characteristics and quality evaluation

The comprehensive meta-analysis encompassed 11 cohort studies spanning a geographical range that included the United States, China, Japan, Korea, France, Spain, and the Netherlands, with publication years extending from 2013 to 2023. The number of children in the eligible included studies ranged from 249 to 1,735, and the mean age of pregnant women across studies was between 26.4 and 32.5 years. A detailed examination of TH levels was predominantly focused on neonatal assessments in seven studies, while three studies focused on early childhood, assessing thyroid function at mean ages of 2, 4, 6, and 10 years. One study uniquely investigated both neonatal and childhood stages, around the age of six years. These studies collectively reported on maternal BPA concentrations, with a subset also exploring exposure to other bisphenols. Notably, the investigations in particular highlighted the exploration of sex-specific effects (**Table 1**).

**Table 1:** Characteristics of Included Studies.

Study Id (First author & Year)	Country	Research Design	Maternal Age	Number of Children			Children’s Age	Type of BPs	Type of THs	Covariates
				Overall	Female	Male				
Chevrier 2013 (1)	USA	Birth Cohort	–	364	176	188	Newborn	BPA	TSH	Mother’s country of birth, and Child’s age.
Romano 2015 (2)	USA	Birth Cohort	–	249	135	114	Newborn	BPA	TSH, TT3, TT4, FT3, FT4	Maternal age, Race, Education, Alcohol consumption, Serum cotinine at 16 weeks gestation, Parity, Prenatal vitamin use, Log -PCB 153, Delivery by Cesarean section, Gestational week at 10 delivery, and Newborn sex.
Minatoya 2017 (3)	Japan	Birth Cohort	30.4±5.0	283	158	127	Newborn	BPA	TSH, FT4	Sex, Interaction of sex and BPA, and Days of mass screening test was conducted.
Derakhshan, 2021 (48)	Netherlands	Birth Cohort	30.5±4.8	Newborn: 853 Children: 882	Newborn: 404 Children: 414	Newborn: 449 Children: 468	Newborn, 5.8-year-old	BPA, BPS, BPF	TSH, FT4	Sex, Fetal distress, Method of delivery Parity, Ethnicity, Maternal education, BMI, Smoking status, Urinary creatinine, Children’s BMI and age.
Jang, 2021 (5)	Korea	Birth Cohort	31.4±3.6	513	243	270	6-year-old	BPA	TSH, TT3, FT4	Age, Sex, BMI, Prenatal age, Prenatal education, Monthly household income, Secondhand smoke, and Urinary creatinine.
Wang 2020 (54)	China	Birth Cohort	–	398	183	215	Newborn	BPA	TSH, FT3, FT4	Urinary creatine concentration, Maternal age, Maternal education, GDM, Husband smoking during pregnancy, Parity, Gestational age at delivery.
Li 2020 (4)	China	Birth Cohort	–	348	151	197	year-old, 4-year-old	BPA	TSH, TT3, TT4, FT3, FT4	Recruitment, Maternal education, Gravidity, Maternal passive smoking, Maternal vitamin supplementation, Child's gender.
Guo 2020 (20)	China	Birth Cohort	26.4±5.7	386	180	206	10-year-old	BPA	TSH, TT3, TT4, FT3, FT4	Maternal age, Maternal education, Pre-pregnancy BMI, Family annual income, Passive smoking during pregnancy, Sex, Creatinine concentration, Ln-transformed analyte concentration × child’s sex.
Sarzo 2022 (55)	Span	Birth Cohort	30.1±4.4	387	190	197	Newborn	BPA	TSH	Sex, and Employment during pregnancy.
Xi 2023 (51)	China	Birth Cohort	–	258	109	1409	Newborn	BPA, BPF, BPAF, BPS, TCBPA	TSH, TT3, TT4, FT3, FT4	Maternal age, Pre-pregnant BMI, Education level, Family income, Parous status, Maternal passive smoking, Type of delivery, Newborn sex, BDE-99 exposure.
Coiffier 2023 (56)	French	Birth Cohort	32.5±3.9	442	237	205	Newborn	BPA, BPS	TSH, TT4	Child age, Sex, Delivery mode, Maternal age, Maternal BMI before pregnancy, Parity, Maternal education, Maternal smoking during pregnancy, Maternal urinary iodine, Serum selenium concentrations and batch.

BPA, Bisphenols A; BPF, Bisphenols F; BPS, Bisphenols S; BPAF, Bisphenols AF; TCBPA, Tetrachlorobisphenol A; TSH, Thyroid stimulating hormone; TT3, Total tri-iodothyronine; TT4, total thyroxine; FT3, Free tri-iothyronine; FT4, Free thyroxine.

The NOS quality assessment results of the studies included are shown in **Table 2**. The NOS scores ranged from 6 to 8 stars, affirming the high quality of the included studies in our analysis, and underscoring the robustness and reliability of the synthesized evidence presented (**Table 2**).

**Table 2:** Study quality of cohort studies.

Study	Selection				Comparability	Outcome			Total score
	Representativeness of the exposed cohort	Selection of the non-exposed cohort	Ascertainment of exposure	Demonstration that outcome of interest was not present at start of study		Assessment of outcome	Was follow up long enough for outcomes to occur	Adequacy of follow up of cohorts	
Chevrier 2013 (1)	*	*	*	*	*	*	–	–	6
Romano 2015 (2)	*	*	*	*	*	*	*	–	7
Minatoya 2017 (3)	*	*	*	*	*	*	*	–	7
Derakhshan 2021 (48)	*	*	*	*	**	*	*	–	8
Jang 2021 (5)	*	*	*	*	**	*	*	–	8
Wang 2020 (54)	*	*	*	*	*	*	*	–	7
Li 2020 (4)	*	*	*	*	*	*	*	–	7
Guo 2020 (20)	*	*	*	*	**	*	*	–	8
Sarzo 2022 (55)	*	*	*	*	*	*	*	–	7
Xi 2023 (51)	*	*	*	*	*	*	*	–	7
Coiffier 2023 (56)	*	*	*	*	*	*	*	–	7

The number of * represents the score.

### 3.3 Primary outcomes: effects of prenatal bisphenols mixtured exposure on thyroid hormones in children of different sex

#### 3.3.1 Prenatal BPs and TSH

**Figure 2:**
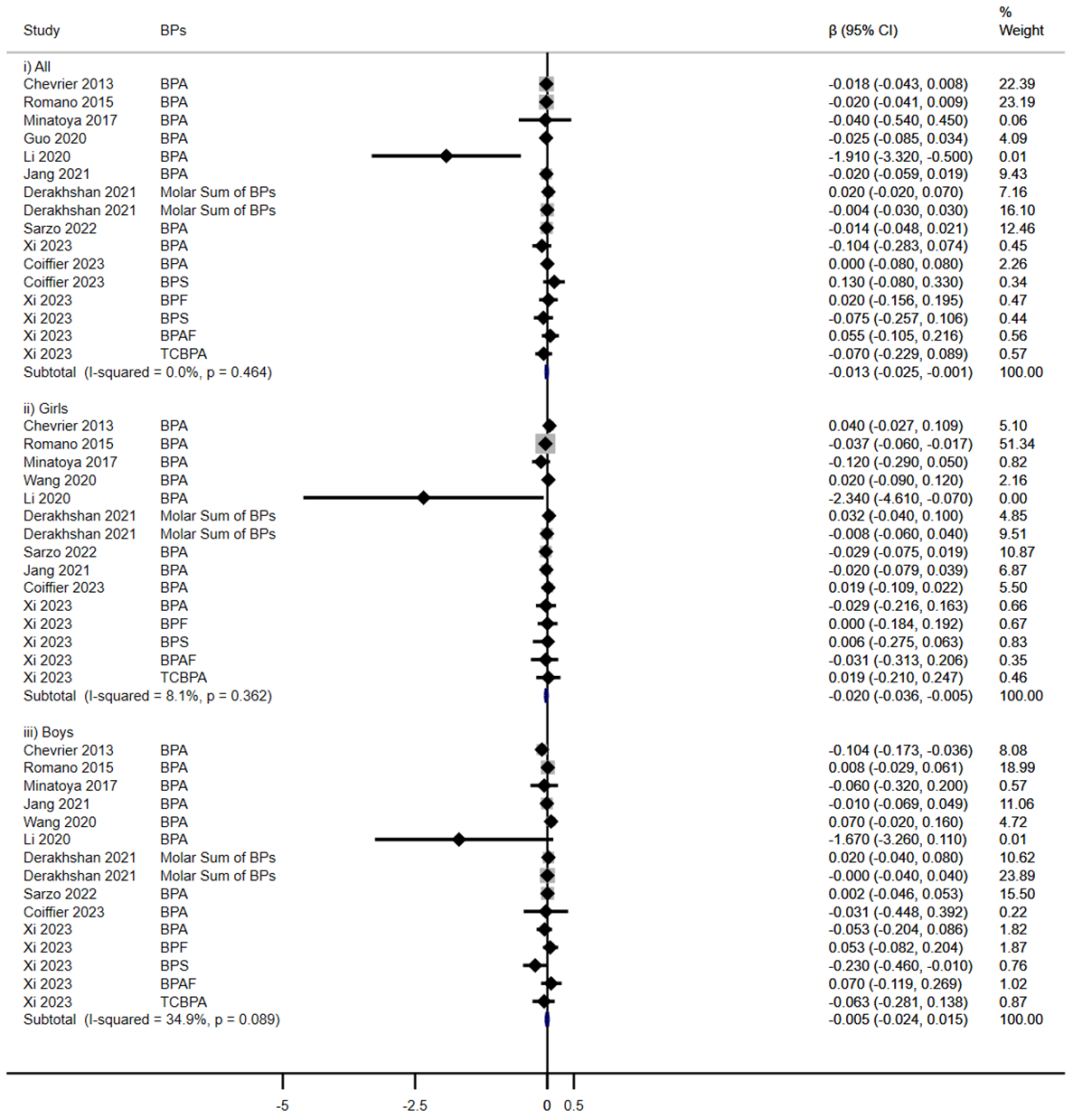
Effect of Prenatal BPs Mixtured Exposure on TSH Levels in Children of Different Sex.

Analysis from eleven cohort studies assessing the impact of prenatal BPs exposure on TSH levels indicated a modest, yet statistically significant reduction in TSH levels in total children (β = -0.013, 95% CI: -0.025, -0.001), a fixed-effects model was employed due to low heterogeneity (I^2^< 50%). Notably, a sex-specific analysis revealed that this reduction was pronounced in female offspring (β = -0.020, 95% CI: -0.036, -0.005), and no significant association was observed in male (β = -0.005, 95%CI: -0.024, 0.015) (**Figure 2**).

#### 3.3.2 Prenatal BPs and TT3

**Figure 3:**
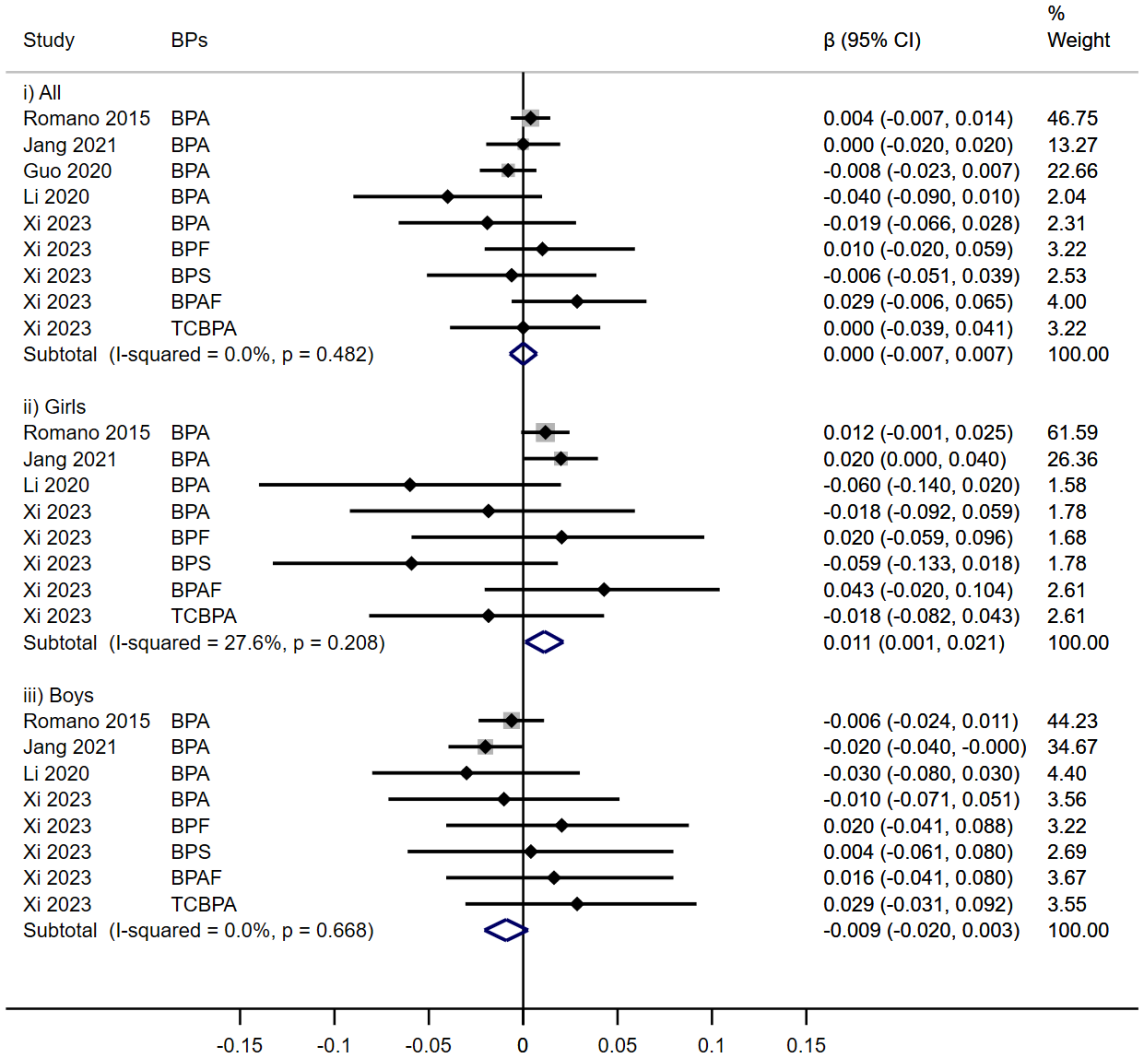
Effect of Prenatal BPs Mixtured Exposure on TT3 Levels in Children of Different Sex.

Five cohorts evaluated the impact of prenatal exposure to BPs on TT3 levels in children (**Figure 3**). In subgroup analyses, a significant increase was noted in females (β = 0.011, 95% CI: 0.001, 0.021), contrary to no discernible effect in overall children (β = 0.000, 95%CI: -0.007, 0.007) or in males (β = -0.009, 95%CI: -0.020, 0.003).

#### 3.3.3 Prenatal BPs and TT4

**Figure 4:**
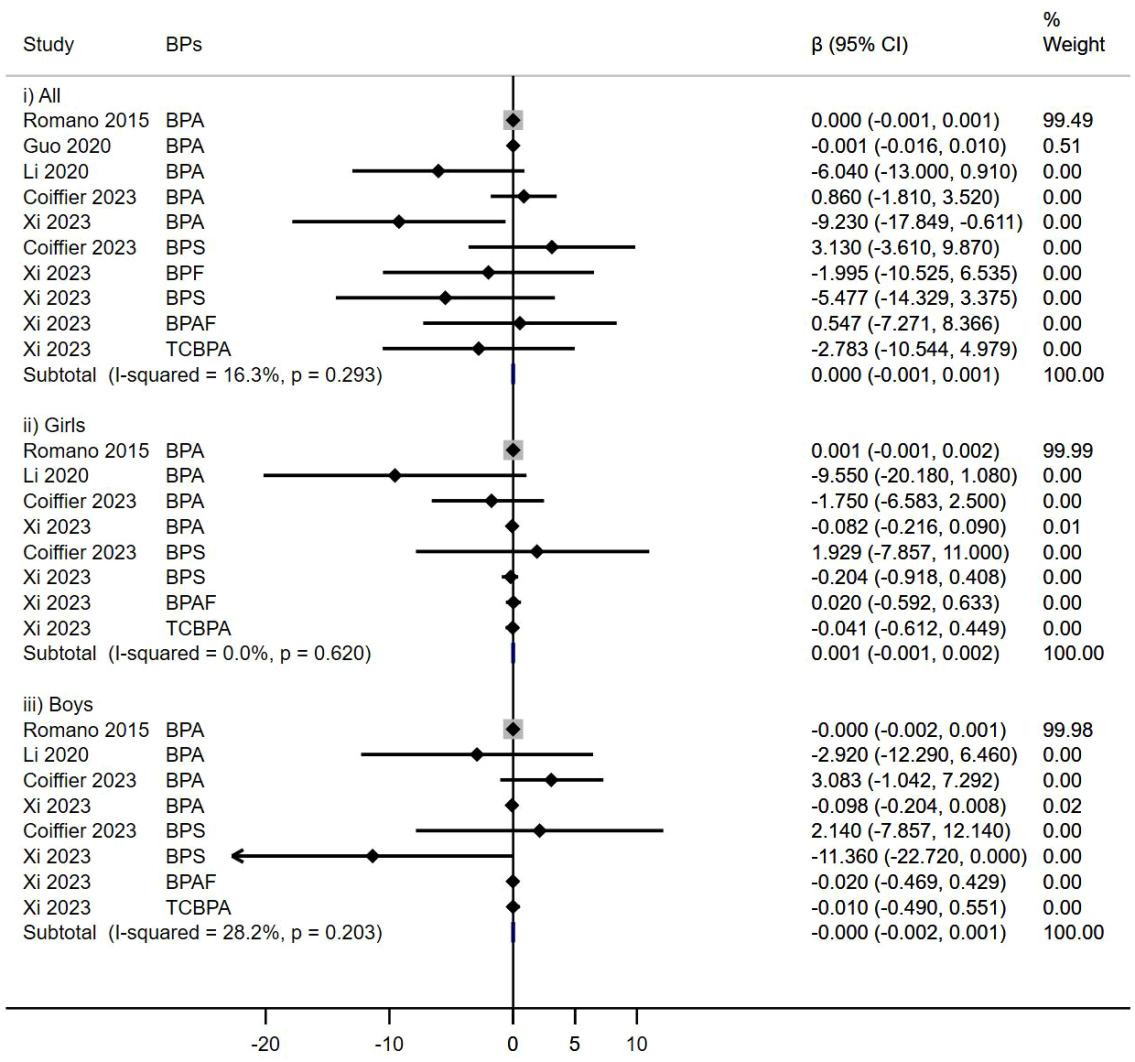
Effect of Prenatal BPs Mixtured Exposure on TT4 Levels in Children of Different Sex.

No significant associations were observed between prenatal BPs exposure and TT4 levels among children across five cohort studies (β = 0.000, 95%CI: -0.001, 0.001), irrespective of sex (Girls: β = 0.001, 95%CI: -0.001, 0.002; Boys: β = -0.000, 95%CI: -0.002, 0.001) (**Figure 4**).

#### 3.3.4 Prenatal BPs and FT3

**Figure 5:**
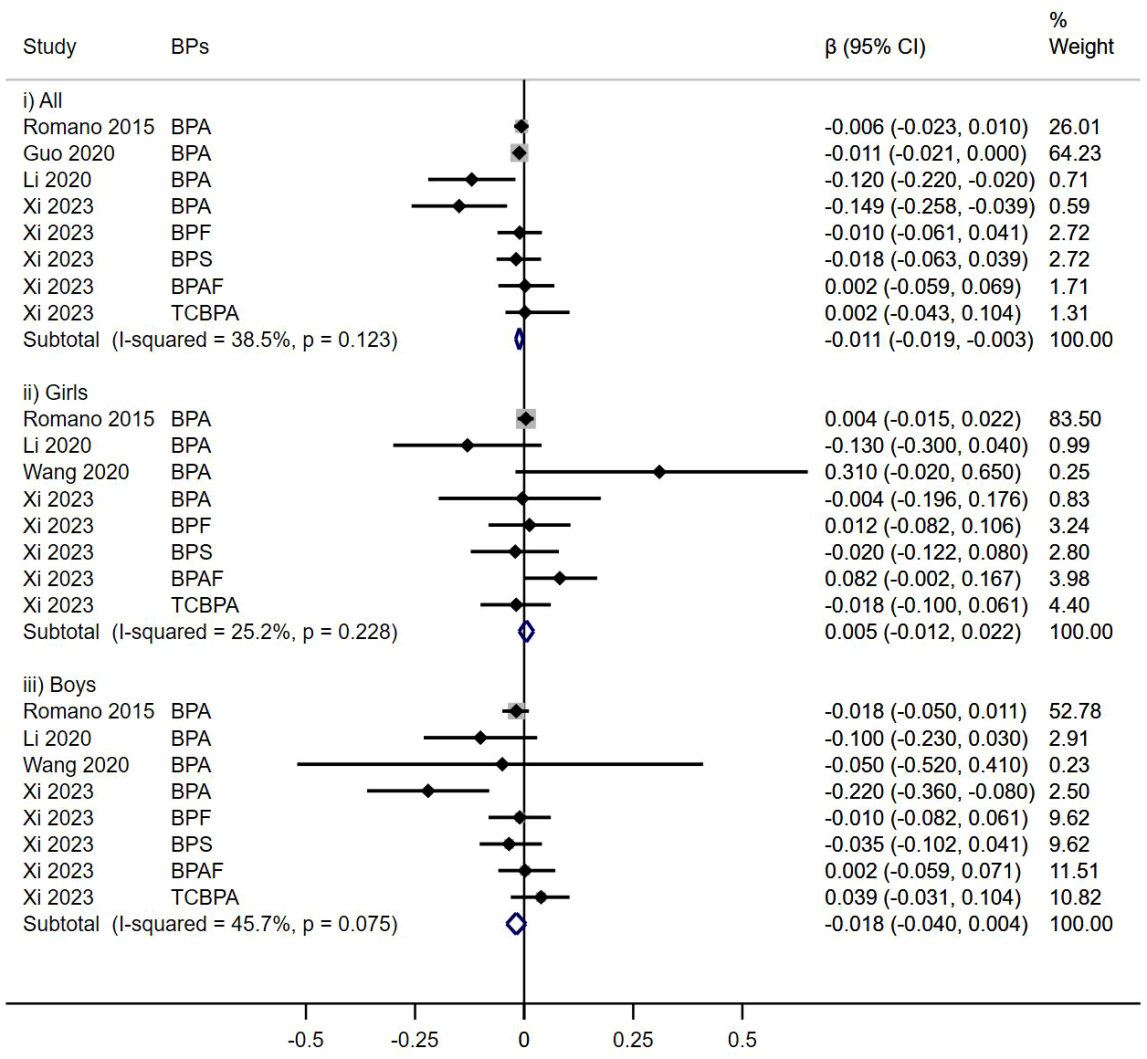
Effect of Prenatal BPs Mixtured Exposure on FT3 Levels in Children of Different Sex.

In this subgroup analysis, prenatal BPs exposure was related to decreased FT3 levels in all children (β = -0.011, 95% CI: -0.019, -0.003). However, this relationship was neither found in females (β = 0.005, 95%CI: -0.012, 0.022) nor males (β = -0.018, 95%CI: -0.040, 0.004) (**Figure 5**).

#### 3.3.5 Prenatal BPs and FT4

**Figure 6:**
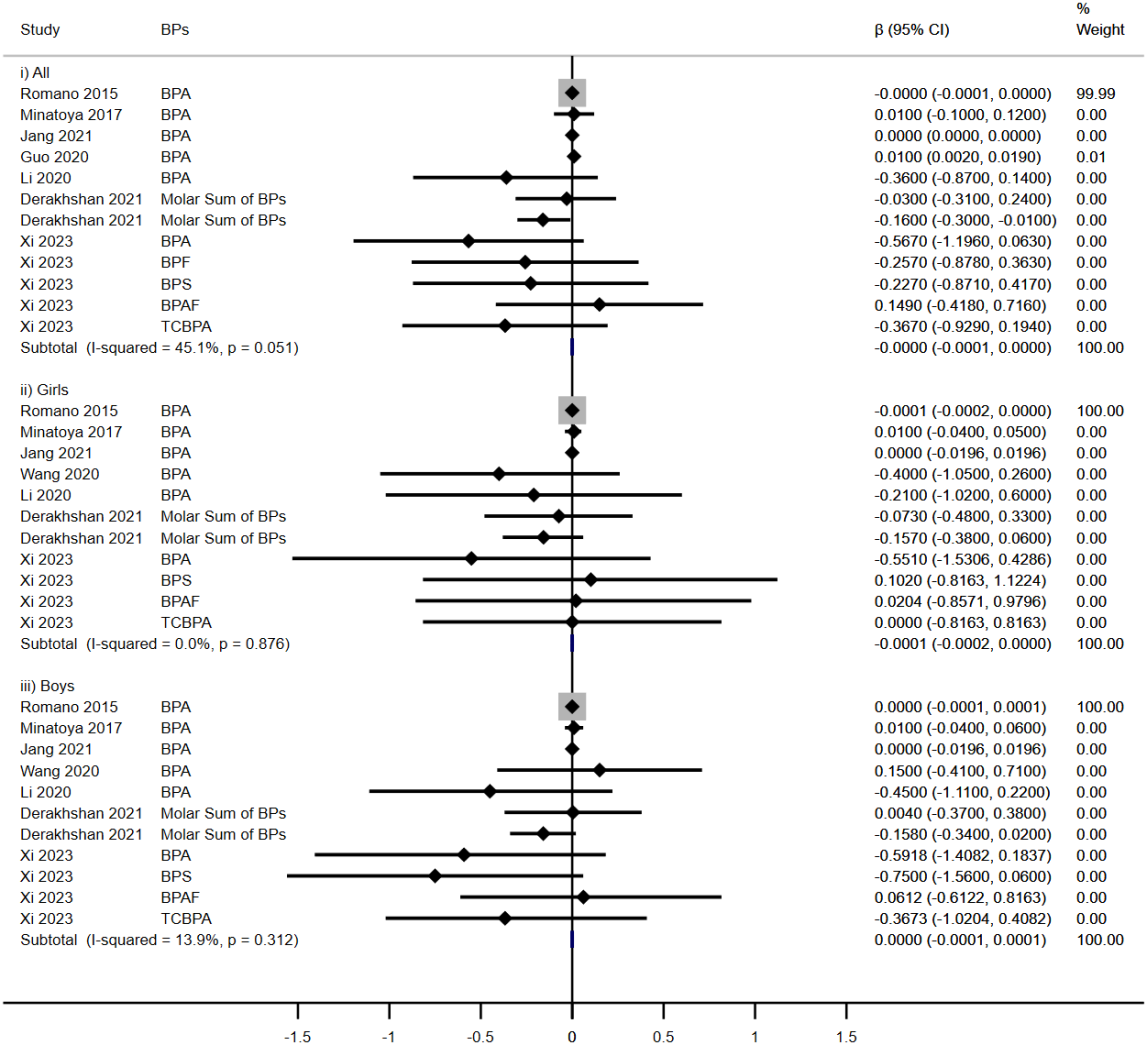
Effect of Prenatal BPs Mixtured Exposure on FT4 Levels in Children of Different Sex.

In an aggregated analysis of eight cohort studies, no significant associations were observed in prenatal BPs exposure and decreased FT4 levels in children (β= -0.00001, 95%CI: -0.0001, 0.0000), irrespective of sex (Girls: β = -0.0001, 95% CI: -0.0002, 0.00001; Boys: β = 0.0000, 95%CI: -0.0001, 0.0001) (**Figure 6**).

### 3.4 Additional outcomes: effects of prenatal BPA exposure on thyroid hormones in children of different sex

Subgroup analysis further elucidated that the observed relationships between prenatal BPA concentrations and alterations in thyroid hormone levels mirrored those identified in the broader bisphenol exposure analysis. Intriguingly, increased TT3 was observed in female offspring (β = 0.012, 95% CI: 0.002, 0.023), and a decrease in TT3 levels was also noted in male offspring (β = -0.013, 95% CI: -0.025, -0.001) (**Supplementary Figures 1A–E**).

### 3.5 Subgroup analysis of the effects of prenatal bisphenols at different exposure levels on thyroid hormones in all children

We established 1.5 ug/g creatinine (Cr) as the threshold value to categorize the exposure groups into high and low levels. Subgroup analysis showed that high levels of bisphenols exposure significantly reduced FT3 levels (β = -0.011, 95% CI: -0.020, -0.003) in contrast to low exposure groups (β = -0.008, 95% CI: -0.037, -0.020), but no specific effects of exposure concentration were found in relation to other thyroid hormones (**Supplementary Figures 2A–E**).

### 3.6 Sensitivity analysis

Sensitivity analysis was conducted to evaluate the stability of the relationship between prenatal bisphenols mixtured exposure and THs in all children, as shown in **Supplementary Figures 3A–E**. The pooled β and 95% CI did not show evident differences after excluding each individual article one by one, indicating the credibility of the studies included in our meta-analysis.

### 3.7 Publication bias

Publication bias in the relationship between prenatal bisphenols mixtured exposure and thyroid hormones in all children was assessed with Egger’s test (**Supplementary Table 2**). Our statistical test showed no evidence of publication bias (Egger’s test p > 0.05).

## 4 Discussion

This meta-analysis investigated the association of maternal bisphenols (BPs) exposure with offspring’s THs levels, and sex differences and exposure levels may change the outcomes. The salient findings are multifaceted: 1) Exposure to BP mixtures notably reduced TSH levels across the pediatric spectrum, and more effects were reported in girls; 2) BPs singularly escalates TT3 levels in female offspring; 3) BPA exposure parallels these effects (reduce TSH levels and increase TT3 levels in girls) while additionally diminishing TT3 levels in boys; 4) High level of BPs mixtured exposure (>1.5 ug/g Cr) could reduce FT3 levels. The detailed results were summarized in **Table 3**.

**Table 3:** Effects of Prenatal BPs on Children by Meta-analyses.

Type of BPs	TSH		TT3		TT4		FT3		FT4	
	β (95%CI)	No. of Cohorts	β (95%CI)	No. of Cohorts	β (95%CI)	No. of Cohorts	β (95%CI)	No. of Cohorts	β (95%CI)	No. of Cohorts
**Bisphenols mixture**	**-0.013 (-0.025, -0.001)**	**10**	0.000 (-0.007, 0.007)	5	0.000 (-0.001, 0.001)	5	**-0.011 (-0.019, -0.003)**	**4**	-0.000 (-0.000, 0.000)	**7**
Subgroup Analyses										
i) Sex										
Girls	**-0.020 (-0.036, -0.005)**	**10**	**0.011 (0.001, 0.021)**	**4**	0.001 (-0.001, 0.002)	4	0.005 (-0.012, 0.022)	4	-0.000 (-0.000, 0.000)	**7**
Boys	-0.005 (-0.024, 0.015)	10	-0.009 (-0.020, 0.003)	4	-0.000 (-0.002, 0.001)	4	-0.018 (-0.040, 0.004)	4	0.000 (-0.000, 0.000)	7
ii) Exposure levels										
Low	-0.018 (-0.042, 0.006)	2	0.010 (-0.010, 0.030)	1	-2.263 (-6.364, 1.838)	1	-0.008 (-0.037, 0.020)	1	-0.170 (-0.467, 0.128)	1
High	-0.012 (-0.026, 0.002)	7	-0.001 (-0.009, 0.006)	5	0.000 (-0.001, 0.001)	4	**-0.011 (-0.020, -0.003)**	**4**	0.002 (-0.012, 0.016)	6
**BPA**	**-0.013 (-0.025, -0.002)**	**10**	-0.001 (-0.009, 0.006)	5	0.000 (-0.001, 0.001)	5	-0.023 (-0.050, 0.004)	4	0.002 (-0.012, 0.016)	7
Subgroup Analyses										
Sex										
Girls	**-0.018 (-0.033, -0.003)**	**10**	0.011 (-0.006, 0.027)	4	0.001 (-0.001, 0.002)	4	-0.002 (-0.102, 0.097)	4	-0.000 (-0.000, 0.000)	7
Boys	-0.009 (-0.028, 0.010)	10	**-0.013 (-0.025, -0.001)**	**4**	-0.000 (-0.002, 0.001)	4	-0.093 (-0.203, 0.016)	4	-0.000 (-0.000, 0.000)	7

BPA, Bisphenols A; BPF, Bisphenols F; BPS, Bisphenols S; BPAF, Bisphenols AF; TCBPA, Tetrachlorobisphenol A; TSH, Thyroid stimulating hormone; TT3, Total tri-iodothyronine; TT4, total thyroxine; FT3, Free tri-iothyronine; FT4, Free thyroxine. Bold values indicate statistical significance.

Prenatal exposure to bisphenols may traverse the placental barrier, potentially exerting long-term effects on fetal thyroid development and function, as well as postnatal life outcomes (21). Therefore, comprehending these impacts is pivotal for pediatric health. Prior studies have indicated that maternal exposure to bisphenols may influence maternal thyroid hormone levels. For instance, urinary BPA has been associated with thyroid function, with elevated concentrations of BPA potentially decreasing levels of TSH (54, 57), TT4 (1, 58), TT3 and FT3 (59) in women. Conversely, exposure to BPB, BPF, and TBBPA (60) may elevate FT4 levels (52), while BPS exposure might lower TT3 levels (52) and elevate TT4 levels (48). Moreover, studies have reported a notable positive trend in serum FT3 and FT4 levels with cumulative exposure to bisphenols mixture (50), although some have suggested that BPA exposure, either single-exposure (48) or in combination with other bisphenols (2, 55), may not correlate with maternal thyroid function. Despite inconsistent findings, the significance of this issue cannot be overstated. A meta-analysis focusing on childhood exposure to bisphenols and pediatric thyroid hormone levels indicated that BPA exposure lowers TSH levels (61). The prenatal period is a critical window for fetal organ and system development. Given the potential window of exposure and the cumulative effects over time, the impact of bisphenol exposure on fetal thyroid function may manifest postnatally. Hence, understanding how maternal exposure to bisphenols influences offspring thyroid function holds crucial implications for preventing and managing pediatric thyroid disorders in public health.

The indispensable role of thyroid hormones in supporting fetal growth, neurological maturation, metabolic regulation, and overall physiological harmony underscores the clinical significance of these findings (4, 62). The contrasting outcomes documented in previous literature (conclusions of the included studies were shown in **Supplementary Table 3**) underscore the intricacy involved in comprehending the association between prenatal exposure to bisphenols and thyroid function. While our study accentuates significant correlations between BPs and THs, disparities with preceding inquiries warrant a meticulous exploration of plausible influencing factors. The diversity within study populations, including demographic characteristics and health status of pregnant women, may significantly influence the outcomes. The study population by Romano et al. was mainly white primiparous women aged 25 - 35 years old from the United States, with higher socioeconomic status and incomes, and usually had regular intake of vitamin pills (2). In contrast, studies by Xi et al. and Li et al., most of the exposed subjects were elderly Asian multipara women who basically did not consume vitamin pills (4, 51). The combined superposition of these factors may be the reason for the differences that Romano et al. observed a relationship between prenatal BPA exposure and reduced TSH levels in female neonates, while the other two studies noted a negative correlation between BPA exposure and TSH levels in male infants. Furthermore, methodological differences, particularly in the sample matrix and the timing and frequency of bisphenols measurement, may contribute to inconsistent results. Minatoy et al. utilized serum samples, with BPA concentrations much lower than those reported in studies using maternal urine samples (3). Furthermore, Sarzo et al. only measured phenolics once in the first trimester (55). Other studies have taken more than one measurement and calculated average concentrations of BPA exposed throughout pregnancy. In addition, the study by Sarzo et al. used a birth cohort in Spain, which gradually restricted or banned the use of BPA as a pollutant (55). Rare relevant literature from Spain shows a decrease in urinary BPA concentrations in pregnant women in 2015-2016 (63) similar to the study by Wang and colleague (54). Phenolics are non-persistent pollutants, and the half-life of BPA is relatively short, and a single measurement may not adequately reflect BPA exposure throughout pregnancy. The lower levels of bisphenols exposure may have contributed to the lack of association between BPA and thyroid parameters observed in studies by Minatoya et al., Sarzo et al., and Wang et al. Another study analyzed BPA concentrations in tertiles indicated that girls in the middle tertile had lower TSH levels, whereas boys in the highest tertile had lower TSH, TT3, and FT3 in cord plasmas. However, BPA exposure did not cause an increase or decrease in TT4 or FT4 (4).

Our study elucidates the relationship between prenatal bisphenol exposure and offspring thyroid hormone levels, particularly highlighting significant differences based on sex, and exposure levels. We found that the mixture of BPs exposure and single exposure to BPA were related to decreased TSH and increased TT3 levels in girls, while only in the case of single BPA exposure, reduced TT3 levels were observed in boys. This observed pattern can be attributed to the structural similarity of BPA to TSH and TT3, as well as its role as an antagonist (43, 64) or agonist of thyroid receptors (65, 66). Specifically, BPA functions as an antagonist to thyroid hormones, disrupting TSH secretion through hormonal feedback mechanisms (67). The sex-specific effects observed in studies on endocrine-disrupting chemicals (EDCs) are reinforced by numerous investigations into the diverse effects of BPA (68, 69). Biological factors for dimorphism include the lower expression levels of uridine diphosphate-glucuronosyltransferase 2B1 (UGT2B1), which is involved in BPA glucuronidation, in males than females (70). Glucuronidation is a crucial step in the metabolism and elimination of phenolic compounds in the body. It involves the conjugation of phenolics with glucuronic acid, rendering them more water-soluble and facilitating their excretion via urine or bile, thereby eliminating phenolic compounds (71, 72). Additionally, the thyroid system exhibits a sexually dimorphic sensitivity to maternal exposure to BPs (70). Gonadal hormones, including estradiol, significantly influence the sex-specific neuroendocrine pathway, impacting the hypothalamic-pituitary-thyroid (HPT) axis, which governs the secretion of thyroid hormones (73). These molecular interactions and physiological responses form a complex network through which bisphenols exert their effects on thyroid hormone levels, revealing the intricacy of endocrine disruption and its varied manifestations between sexes. This meta-analysis also revealed that exposure to a mixture of bisphenols exceeding 1.5 µg/g creatinine is associated with a decrease in FT3 levels. A study found a significant correlation between high-dose bisphenol exposure and thyroid function alterations, whereas exposure at low doses may not have a significant impact (74). Similarly, another study noted that the effects of bisphenols on thyroid function may exhibit dose-dependent effects (35). Furthermore, at low concentrations, bisphenols may not adequately bind to thyroid receptors, leading to receptor saturation. In contrast, high-concentration bisphenol exposure may increase the binding affinity of bisphenols to thyroid hormone receptors, thereby intensifying interference with thyroid function. Additionally, exposure to bisphenols at lower concentrations may be more readily metabolized and excreted by the body, reducing their accumulation and duration in the body.

Further to these pathways, bisphenols may affect the transcriptional expression by altering transcription-related genes (Foxe1, Pax8, Nkx2-1) and thyroid hormone synthesis genes (Tpo, Tg, Slc5a5), with bisphenol analogs BPAF and BPS influencing transcriptional changes at doses lower than BPA (75, 76). BPA has the potential to hinder iodine uptake by interacting with the sodium/iodide symporter (NIS) (77, 78), a crucial mediator of iodine transport in thyroid and extrathyroidal tissues, thereby impacting thyroid function (75, 79). NIS plays a crucial role in transporting iodine from the bloodstream into thyroid cells, a pivotal step in thyroid hormone synthesis (80, 81). NIS is responsible for transporting iodine into thyroid cells, which is essential for the synthesis of thyroid hormones, particularly thyroxine (T4) (82, 83). Additionally, since TSH stimulates the thyroid to release T4, compensatory elevation of TSH levels may occur (84). Another possible mechanism is that their ability to interact with estrogen and androgen receptors (85). Animal studies provide evidence that BPA impacts hormone synthesis in the pituitary gland, affecting TSH production through estrogen receptor signaling (86–89), independently of thyroid hormone feedback loops. These studies have reported varying results, possibly attributable to differences in the chemical structures tested, dosages used, and species studied. Further *in vivo* controlled experiments are needed to elucidate the extent and mechanisms of BP influence on NIS and thyroid hormone levels. The mechanisms of action of bisphenol compounds were shown in **Supplementary Table 4**.

This study is the first meta-analysis to investigate the effect of maternal BPs exposure on thyroid hormone levels in children and explore the sex-specific effect on thyroid function, and incorporated studies were high-caliber prospective cohort investigations, defined by explicit search criteria, potentially reinforcing the trustworthiness of our research. However, it has certain limitations. First, studies were interpreted in different subgroup analyses, such as types of exposure and THs, and children’s sex. These subgroup analyses demonstrated the potential directions of further public health concerns, but, unfortunately, the analyses were limited by a relatively small sample size, we were unable to analyze the single effects of other bisphenol analogs on thyroid hormones. Second, studies used different types of outcome measurements, although we conducted sensitivity analyses on individual tests, substantial heterogeneity across the included studies was not fully interpreted. Third, most of the research subjects were neonates, but there were only four studies focused on early childhood, which may lead to imprecise results. Finally, the included cohort studies were mainly from North Americans and Asians, and there is a dearth of relevant studies in other regions, potentially leading to geographic bias.

## 5 Conclusion

Prenatal exposure to bisphenols reduced TSH and increased TT3 levels in female offspring. Maternal BPA exposure showed an inverse relationship with TT3 levels in male offspring. Notably, high concentrations of bisphenol exposure have also been found to decrease FT3 levels in offspring. Given these findings, it is critical to strengthen control over prenatal exposure to bisphenols to safeguard early childhood health. To validate our findings, a larger and broader prospective cohort study should be conducted to explore the effects of prenatal bisphenols exposure on thyroid hormone levels in children.

## Data availability statement

The original contributions presented in the study are included in the article/**Supplementary Material**. Further inquiries can be directed to the corresponding authors.

## Author contributions

JL: Data curation, Investigation, Methodology, Visualization, Writing – original draft, Writing – review & editing. MT: Data curation, Investigation, Writing – review & editing. HQ: Writing – review & editing. DC: Writing – review & editing. SM: Writing – review & editing. XW: Resources, Supervision, Writing – review & editing. FB: Resources, Supervision, Writing – review & editing.
