# Supplementary material for: Maternal bisphenols exposure and thyroid function in children: a systematic review and meta-analysis
Source: Front Endocrinol (Lausanne). 2024 Jul 1;15:1420540. doi: 10.3389/fendo.2024.1420540 (PMC11246848; doi:10.3389/fendo.2024.1420540)
Supplement: Supplementary file 1 [file DataSheet_1.docx]

**Supplementary Tables:**

**Supplementary Table 1. Search Strategies**

| 1. gestation OR pregnancy OR prenatal OR antepartum OR maternal |
| --- |
| 1. BPA OR BPF OR BPS OR bisphenol |
| 1. child OR kid OR ped OR neonatal OR neonate OR baby OR newborn OR infant OR boy OR girl |
| 1. thyroid OR TSH OR TT3 OR TT4 OR FT3 OR FT4 OR total triiodothyronine OR total thyroxine OR free triiodothyronine OR free thyroxine OR thyroid-stimulating hormone OR thyroid disease OR hyperthyroidism OR subclinical hyperthyroidism OR hypothyroidism OR subclinical hypothyroidism |
| 1. 1+2+3+4 |

**Supplementary Table 2. Egger’s Test of Publication Bias in the Relationship between Prenatal BPs Mixtured Exposure and THs in All Children.**

| Type of THs | P > \| t \| |
| --- | --- |
| TSH | 0.467 |
| TT3 | 0.585 |
| TT4 | 0.164 |
| FT3 | 0.177 |
| FT4 | 0.228 |

**Supplementary Table 3. The Statistically Significant Associations of BPs Exposure with THs.**

| Study | Outcomes |
| --- | --- |
| Chevrier 2013 | Inverse associations between maternal BPA urine concentrations and TSH in males, but not female. |
| Romano 2015 | Inverse relationship between late pregnancy maternal BPA concentrations and TSH in female newborns. |
| Minatoya 2017 | No correlation. |
| Derakhshan 2021 | A higher late pregnancy maternal BPA exposure was associated with a higher TSH in female newborns and a higher FT4 during childhood in males. |
| Jang 2021 | Inverse relationship between maternal BPA concentrations and TT3 in all children, and lower FT4 levels were associated with higher urinary BPF concentrations in girls only. |
| Wang 2020 | No correlation. |
| Li 2020 | Girls in the middle tertile BPA concentrations had lower TSH levels, whereas boys in the highest tertile BPA concentrations had lower TSH, and inverse associations between BPA and TT3, FT3. |
| Guo 2020 | Prenatal urinary BPA concentrations were associated with increased FT4. |
| Sarzo 2022 | No correlation. |
| Xi 2023 | Gestational BPA exposure was associated with decreased TT4 and FT3, and BPS exposure was associated with decreased TSH in boys. The mixture of five BP exposures presented a significant positive association with TT3. |
| Coiffier 2023 | No correlation. |

**Supplementary Table 4. Mechanisms of Action of Bisphenol Compounds.**

| Type of BPs | Mechanisms of action |
| --- | --- |
| BPA | 1. Altering the expression of genes related to thyroid hormone synthesis, metabolism and action; 2. Inhibiting the activity of the sodium-iodide symporter (NIS). When BPA inhibits NIS, it reduces the uptake of iodide into thyroid cells, leading to decreased synthesis of thyroid hormones; 3. Alter the pituitary-thyroid axis homeostasis, alter the gene expression of the thyroid hormone receptor β and proteins necessary for thyroid hormone synthesis. |
| BPF/BPS | 1. Exerting estrogenic, antiestrogenic, androgenic, and antiandrogenic effects; 2. Potency in aryl hydrocarbon activity and inhibitory hormonal signaling in adipocytes. |

**Supplementary Figures:**

**Supplementary Figure 1. Effect of prenatal BPA Exposure on THs Levels in Children of Different Sex**

**a. Effect of Prenatal BPA Exposure on TSH Levels in Children of Different Sex**


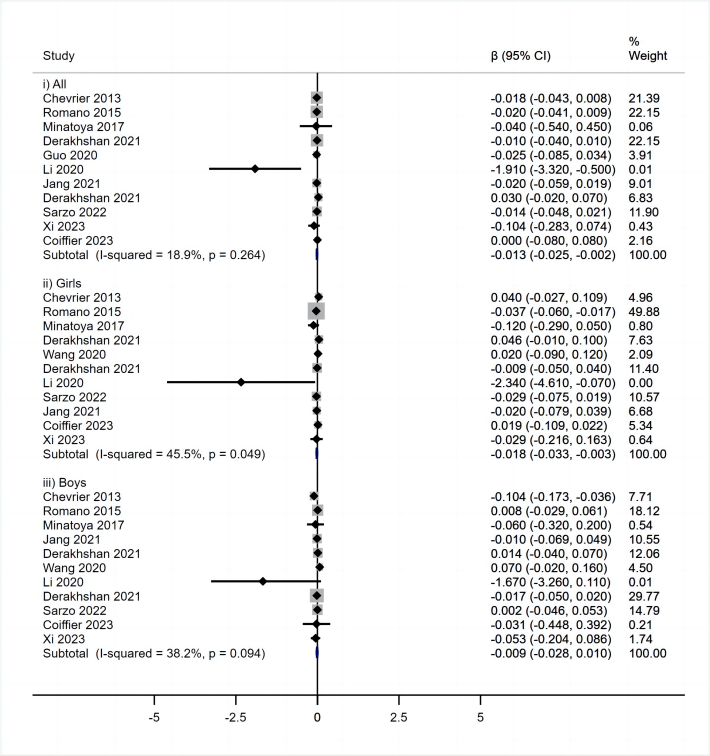


**b. Effect of Prenatal BPA Exposure on TT3 Levels in Children of Different Sex**

**
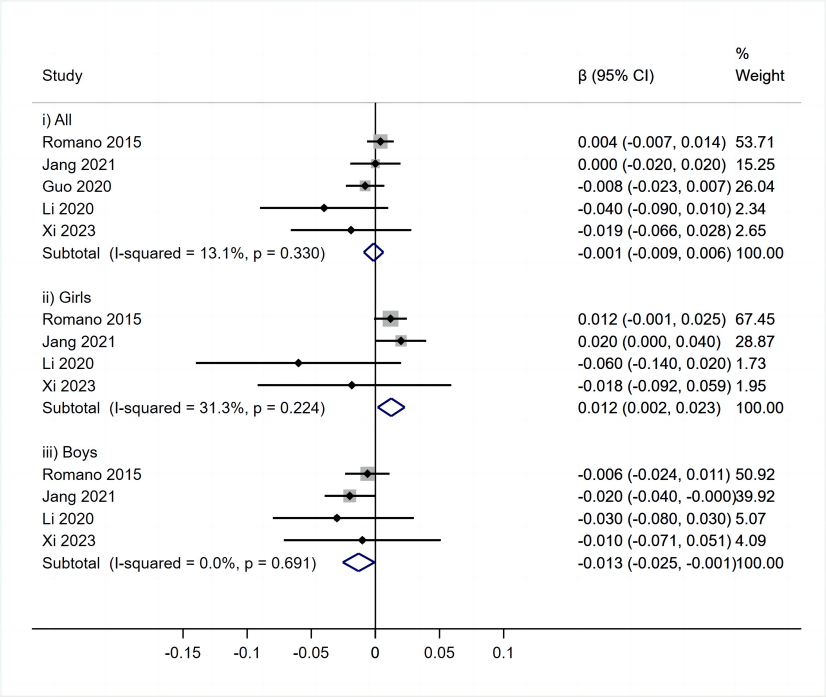
**

**c. Effect of Prenatal BPA Exposure on TT4 Levels in Children of Different Sex**

**
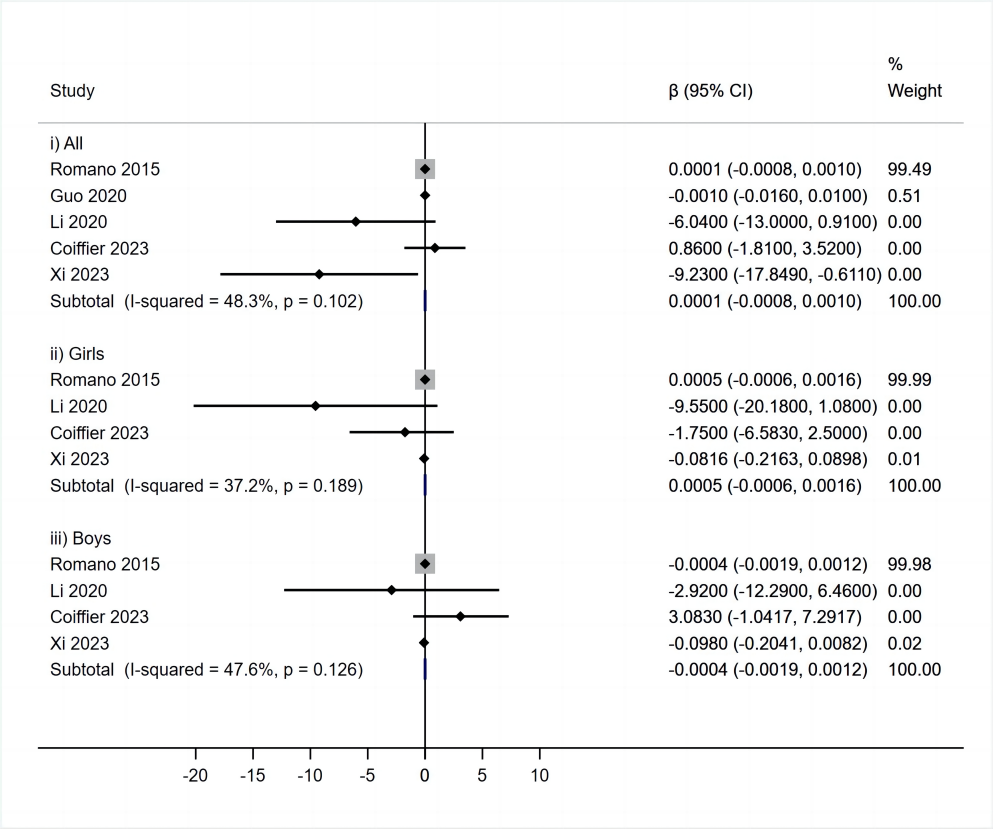
**

**d. Effect of Prenatal BPA Exposure on FT3 Levels in Children of Different Sex**

**
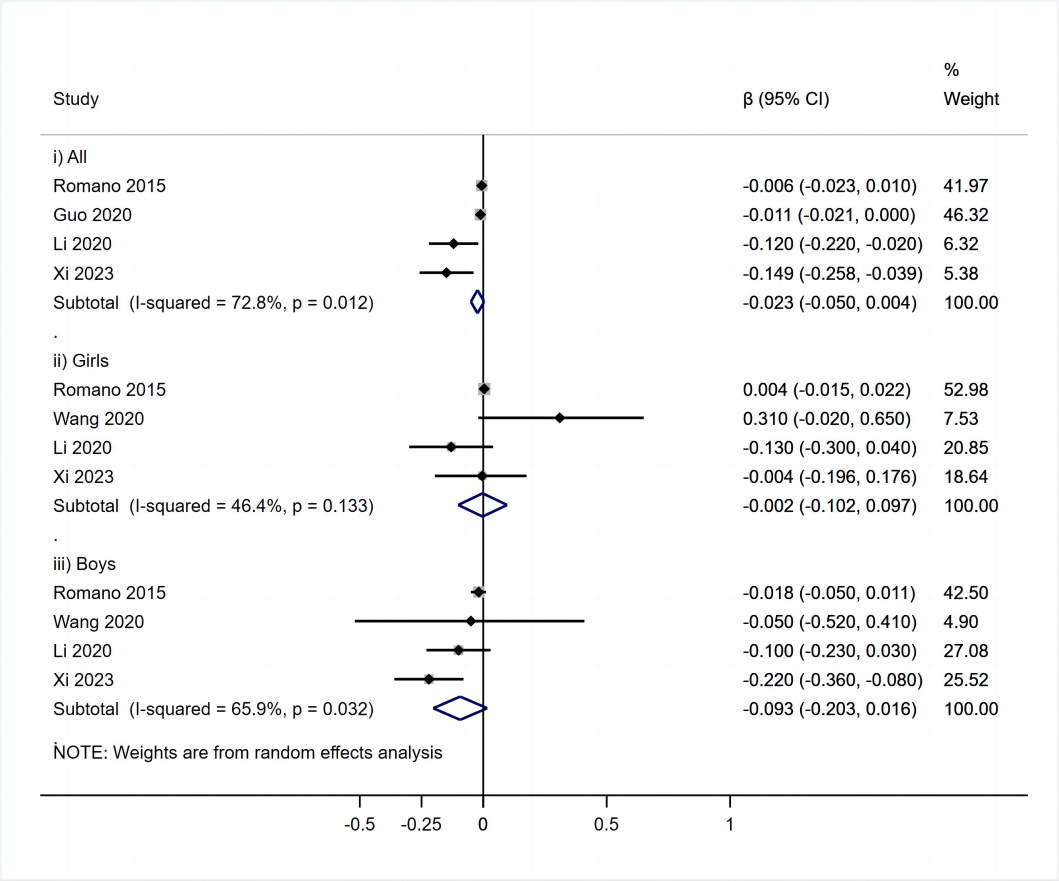
**

**e. Effect of Prenatal BPA Exposure on FT4 Levels in Children of Different Sex**

**
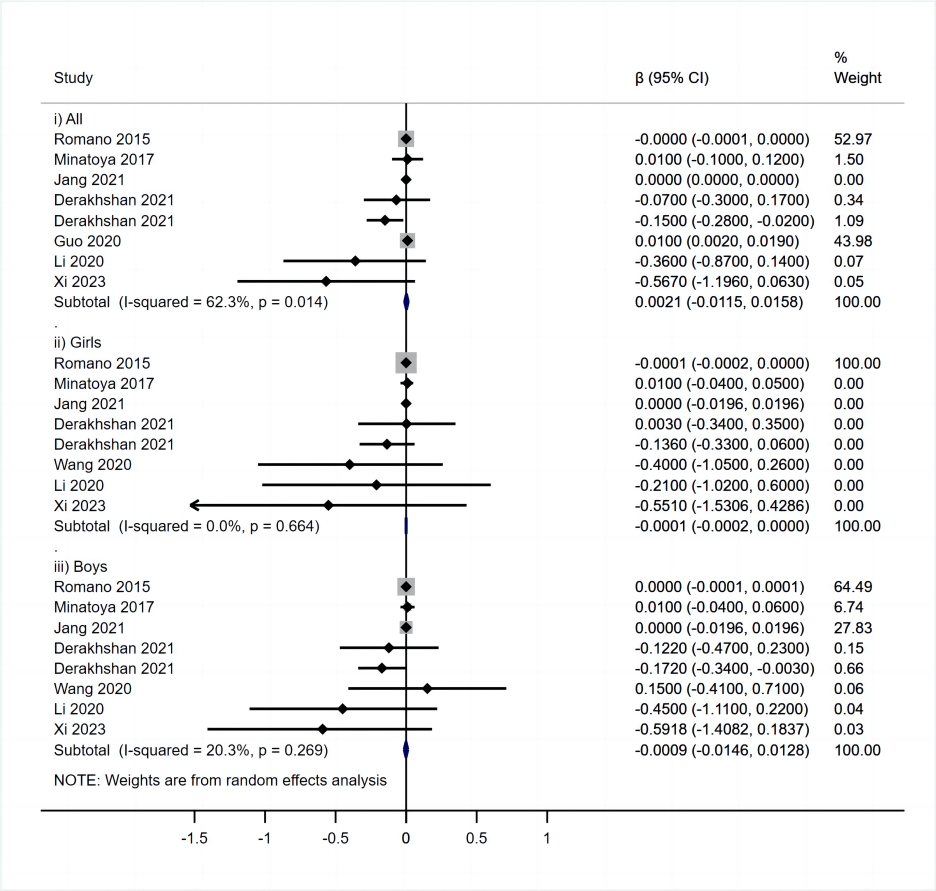
**

## **Supplementary Figure 2. Subgroup Analysis of BPs Exposure Levels**

## **Effect of Prenatal BPs at Different Exposure Levels on TSH in All Children**

**
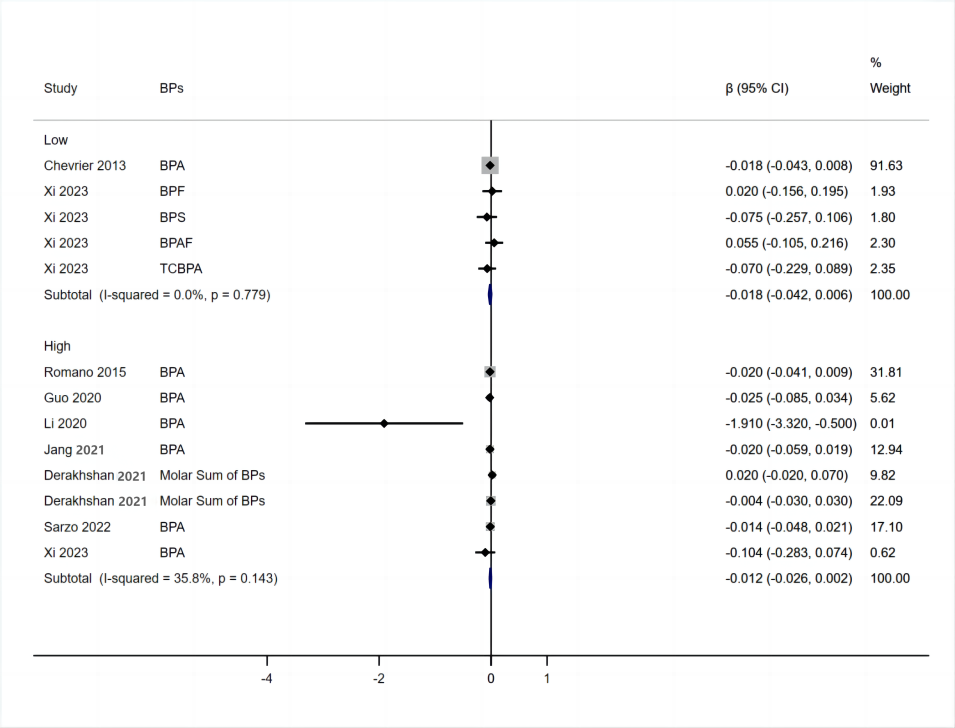
**

## **Effect of Prenatal BPs at Different Exposure Levels on TT3 in All Children**

**
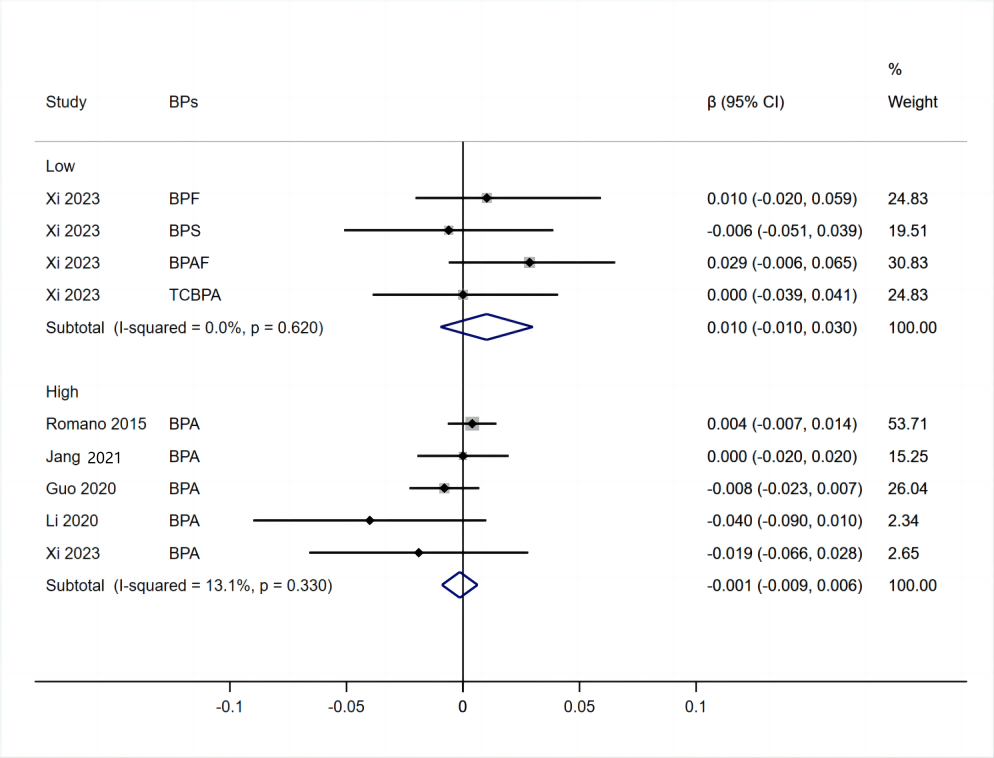
**

## **Effect of Prenatal BPs at Different Exposure Levels on TT4 in All Children**

**
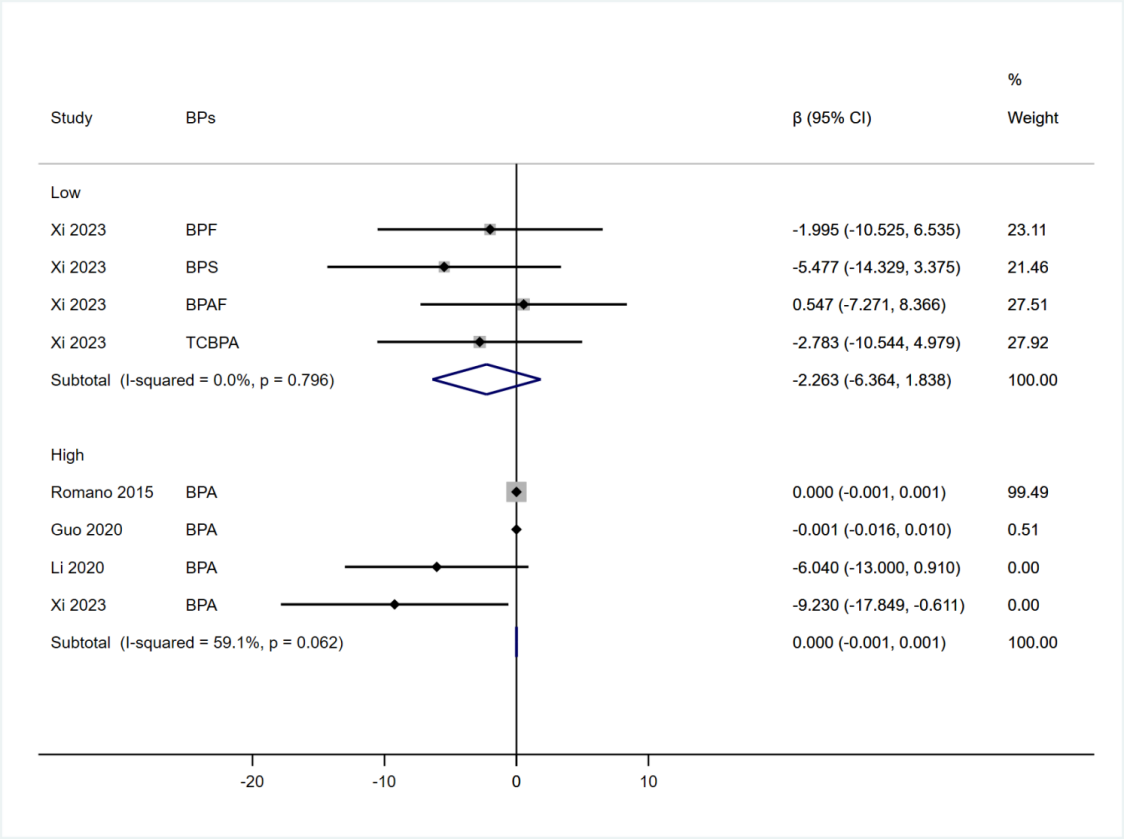
**

## **Effect of Prenatal BPs at Different Exposure Levels on FT3 in All Children**

**
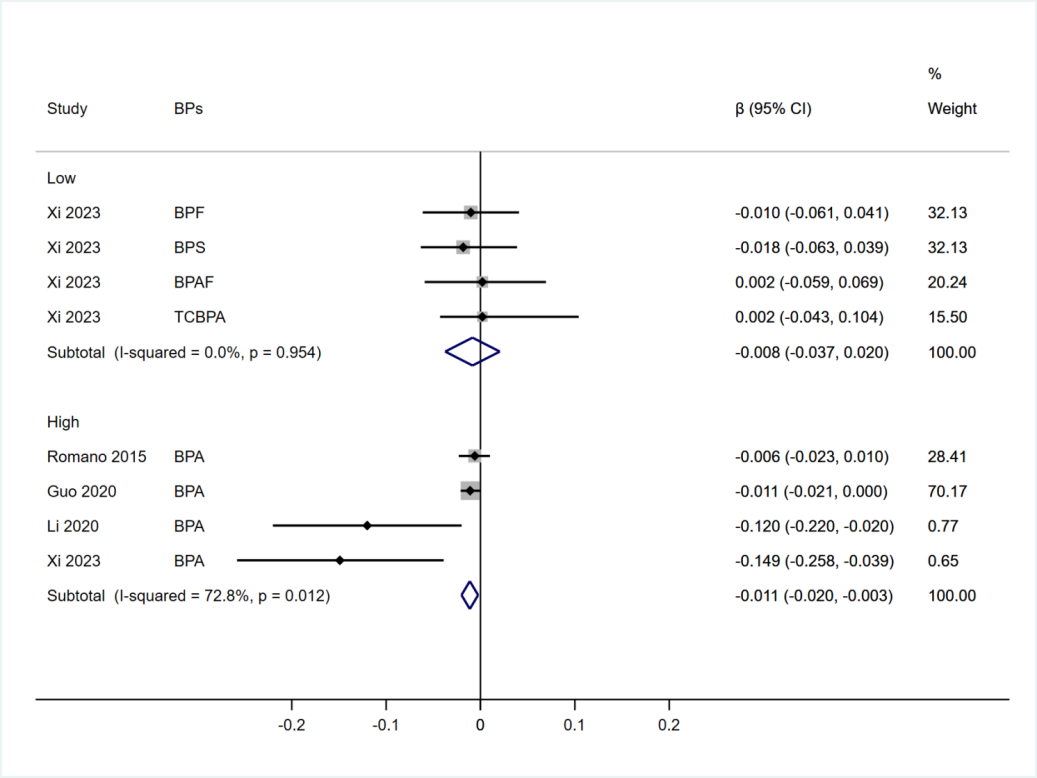
**

## **Effect of Prenatal BPs at Different Exposure Levels on FT4 in All Children**

**
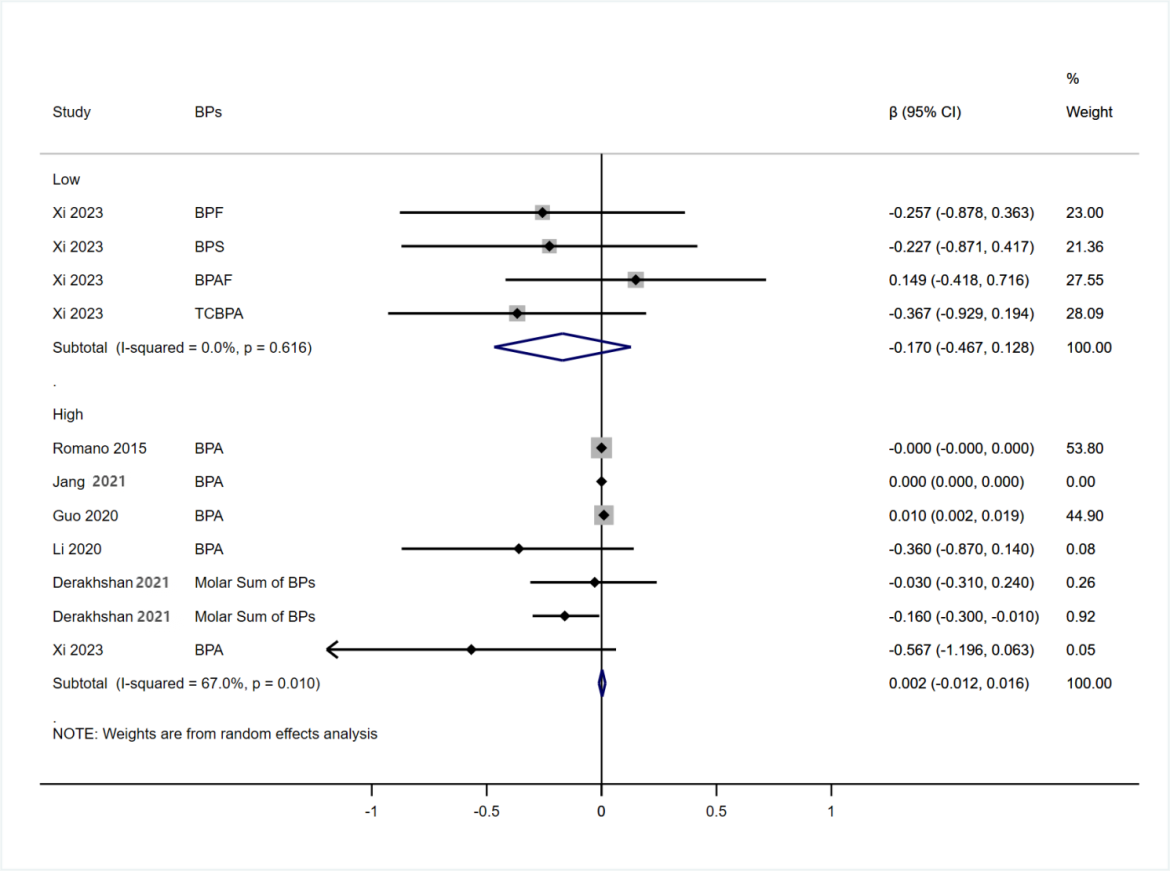
**

**Supplementary Figure 3. Sensitive Analyses of Prenatal BPs Exposure on THs Levels in All Children**

1. **Sensitive Analyses of Prenatal BPs Exposure on TSH Levels in All Children**

1. **Sensitive Analyses of Prenatal BPs Exposure on TT3 Levels in All Children**

1. **Sensitive Analyses of Prenatal BPs Exposure on TT4 Levels in All Children**

1. **Sensitive Analyses of Prenatal BPs Exposure on FT3 Levels in All Children**

1. **Sensitive Analyses of Prenatal BPs Exposure on FT4 Levels in All Children**
